# Applying transformer-based deep learning models in image-driven cancer diagnosis: a comprehensive bibliometric analysis of global research trends

**DOI:** 10.3389/fonc.2026.1592750

**Published:** 2026-05-18

**Authors:** Liyue Gong, Tingxiao Wen, Xu Zeng, Xiaoying Liu, Mengdan Li, Jianwei Sun, Jing Zheng

**Affiliations:** 1 Library, Hunan University of Chinese Medicine, Changsha, China; 2 Shenzhen Health Development Research and Data Management Center, Shenzhen, Guangdong, China; 3 School of Life Sciences, Central South University, Changsha, China; 4 Library, Central South University, Changsha, China

**Keywords:** deep learning, diagnosis, image, oncology, transformer

## Abstract

**Background/objectives:**

The transformer, introduced in 2017, has revolutionized several fields, including oncology. Its applications in cancer detection, diagnosis, prognosis prediction and treatment have shown significant potential. However, a comprehensive analysis of the global research trends and future directions in image-driven cancer diagnosis is lacking.

**Methods:**

We conducted a bibliometric analysis using CiteSpace and VOSviewer to explore the development of transformer applications in image-driven cancer diagnosis(TICD). A total of 2923 papers published between 2017 and 2026 were analyzed, with early access papers from 2026 included. We examined publication trends, international collaboration, and citation patterns to identify research hotspots and emerging directions.

**Results:**

The number of publications on transformer applications in image-driven cancer diagnosis has rapidly increased, with a notable surge beginning in 2022. China and the United States are the leading contributors to the field, with high levels of international collaboration. The primary research focuses on the application of transformer-based models for image classification, segmentation, and enhancement. Their development is moving toward lightweight design, interpretability, multimodal fusion, and low annotation dependence. However, while the volume of publications is increasing, the impact (measured by citation counts) varies across countries and institutions.

**Conclusions:**

The field of TICD is in a robust growth phase, attracting significant attention from global researchers, particularly from China and the United states. While international collaboration is prevalent, the field faces challenges regarding the generalizability and scalability of research findings. Future research should focus on translating these promising technologies into clinical practice, ensuring that they are adaptable and applicable in diverse oncology contexts.

## 1 Introduction

With the rapid development of artificial intelligence (AI) technology, the integration of AI and medicine has become one of the most promising innovative fields (1, 2). Deep learning, as the dominant method in AI, has widespread applications in oncology (3). The Transformer, introduced by Google’s research team in 2017 (4), utilizes multi-head self-attention mechanisms and position encoding to enable efficient parallel computation and capture broader dependencies. It has demonstrated exceptional performance and significant potential in tumor diagnosis (5) and prediction (6).

Accurately identifying tumor-related regions in medical images is crucial for tumor staging, treatment planning, and surgical navigation, with significant clinical value. The Transformer architecture aids tumor diagnosis by enhancing medical imaging (7, 8), medical image segmentation (9–11), and multimodal classification (12–14). It also predicts tumor growth using biomarkers (15) and builds survival models to forecast prognosis (16–18), providing essential decision support for precision medicine and personalized treatment plans.

Transformers have broken through the limitations of traditional methods in tumor image tasks, achieving superior performance in diagnosis and classification (19), prognosis prediction (20, 21), and cross-center generalization (22).The development of TICD represents an important direction for medical progress.Tracking advances in this field is of great significance for promoting medical research and facilitating clinical translation.

Many scholars have conducted research on the aforementioned issues, with some focusing on specific areas, such as the use of transformers in brain tumor image segmentation, precise tumor diagnosis, or the diagnosis and prediction of specific cancers (e.g., breast cancer, lung cancer) from a systematic review perspective (23–26). However, a macro-level bibliometric analysis of the overall research situation has not been conducted. The advancement of TICD remains an unexplored frontier in contemporary scholarly inquiry. With the advancement of large models, the Transformer architecture will no longer be confined to a single type of cancer or a single function; instead, it is likely to make significant contributions to multimodal large models and pan-tumordiagnosis. Therefore, a comprehensive review of various research directions of TICD will help provide references for future developments. The temporal relevance of research constitutes a critical factor that scholars must prioritize in the production of academic outcomes, as the imperative to rapidly comprehend disciplinary landscapes and monitor emergent scholarly developments presents a significant challenge within contemporary academia. Bibliometrics can objectively quantify and analyze research progress, trends, evolution, and the distribution of research efforts in a particular field, offering a comprehensive understanding of its current state. This paper aims to reveal the application status and development trends of TICD from aspects such as publication trends, international collaboration, and thematic evolution, with the results presented in a visual format. This study helps scholars quickly and intuitively grasp the research landscape, track progress, and identify emerging hotspots in the field, thereby advancing the development and innovation of intelligent diagnostic imaging for cancer.

## 2 Materials and methods

### 2.1 Data source

We selected the Science Citation Index Expanded (SCI-EXPANDED) from the Web of Science Core Collection as the data source. The search query was “Topic=(“attention mechanism” OR “transformer” OR ViT OR BERT OR GPT OR “Self-attention network” OR “Multi-head attention”) AND Topic=(Tumor or Neoplasms or Oncology or Cancer) AND Topic= imag*” with the search conducted on December 29, 2025. Since the Transformer architecture was first proposed in 2017, we set the publication period from 2017 to the present. To capture the latest developments in the field, we included early access articles. Early Access publications were identified using the standardized “Early Access” document type label provided in the Web of Science Core Collection metadata. All Early Access records were included in the main analysis to ensure a comprehensive and up-to-date bibliometric overview. Therefore, the inclusion criteria were: (a) article, early access, proceeding paper and review article types; (b) literature published between 2017 and 2026; (c) literature published in English. (d)Topic-relevant publications. After manual screening and verification, 1264 insufficiently relevant publications were excluded. These primarily include medical image processing studies adopting CNN algorithms related to attention mechanisms, where the Transformer architecture was not utilized. Two scholars from relevant fields selected 200 documents to assess their eligibility for inclusion criteria, with a Cohen’s Kappa of 77.5%.

**Figure 1:**
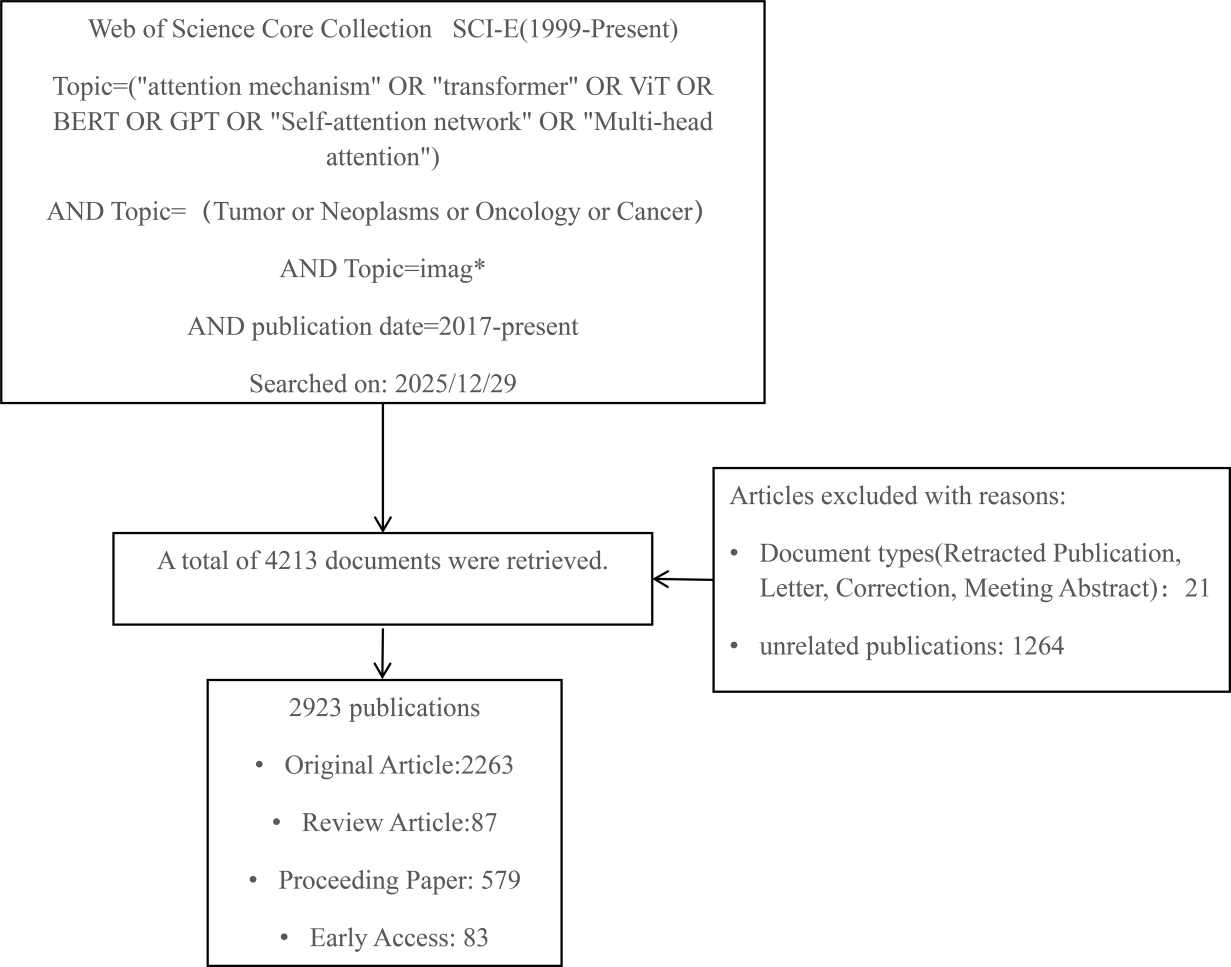
Flowchart of literature screening.

The process of literature acquisition and selection is shown in **Figure 1**.

### 2.2 Bibliometric analysis

We used Microsoft Excel 2019 for flowcharts and statistical tables. Charticulator and SCImago Graphica 1.0.25 were employed for analyzing and visualizing international research collaborations. For bibliometric analysis of authors, journals, institutions, keywords, references, and citations, we utilized Citespace 6.4.R1 and VOSviewer 1.6.20. Citespace and VOSviewer are widely used tools for bibliometric analysis and visualization, enabling the identification of research hotspots and trends across various disciplines (27–29).

## 3 Results

### 3.1 General data

Based on the literature retrieval strategy, a total of 2923 papers published in SCI-E since 2017 were collected, including 2263 research articles and 87 review articles. These publications were authored by 13357 researchers from 3328 institutions across 97 countries/regions and were published in 818 journals.

### 3.2 Publication trend

**Figure 2:**
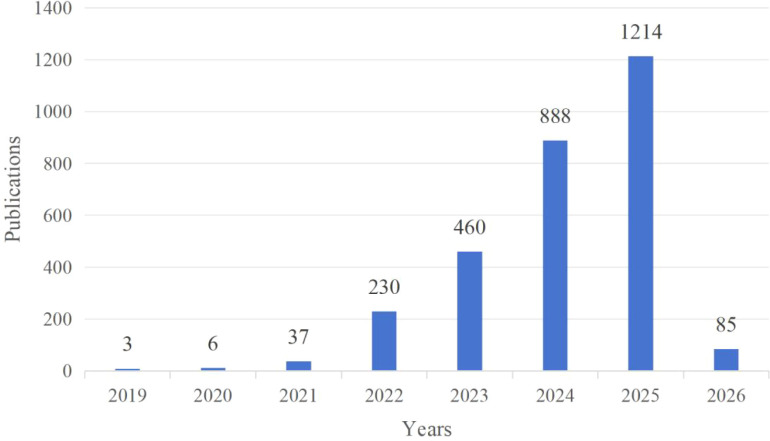
Publication trend of TICD.

From 2019 to 2026, the number of publications gradually increased, with a sharp surge in 2022, reaching a growth rate of 100%. Although the upward trend continued thereafter, the growth rate declined (**Figure 2**). As of the retrieval date, 2025 had the highest number of publications, accounting for 30.38% of the total. Additionally, 85 early access papers for 2026 had already been published.

**Figure 3:**
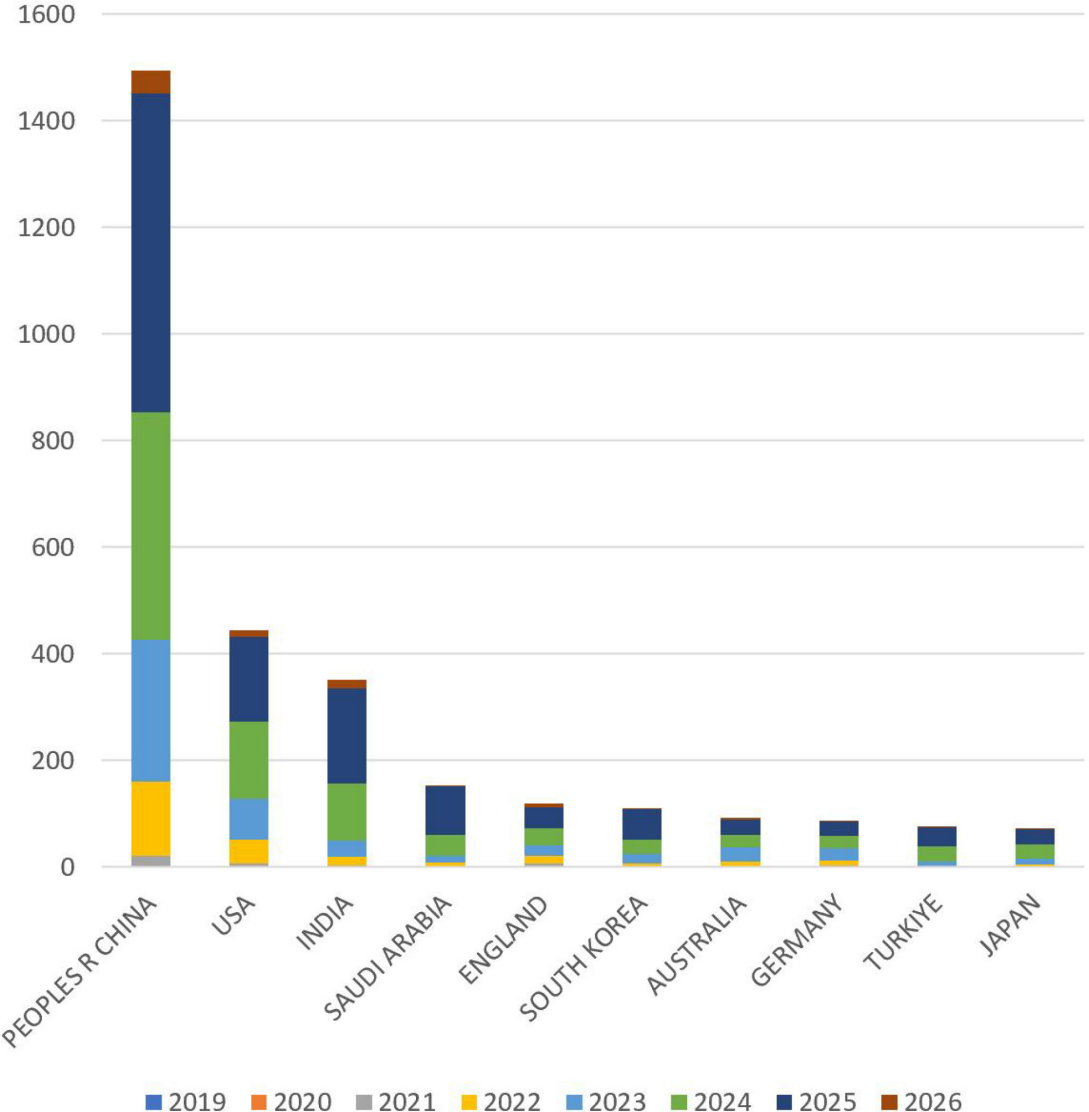
Top 10 countries/regions with annual publication trends of TICD.

The publication trends of the top 10 publishing countries are shown in **Figure 3**. Most countries experienced a surge in publications in 2024, maintaining an upward trend. However, The growth rate of the number of publications in Australia and Germany has slowed down.

### 3.3 Analysis of research countries

**Figure 4:**
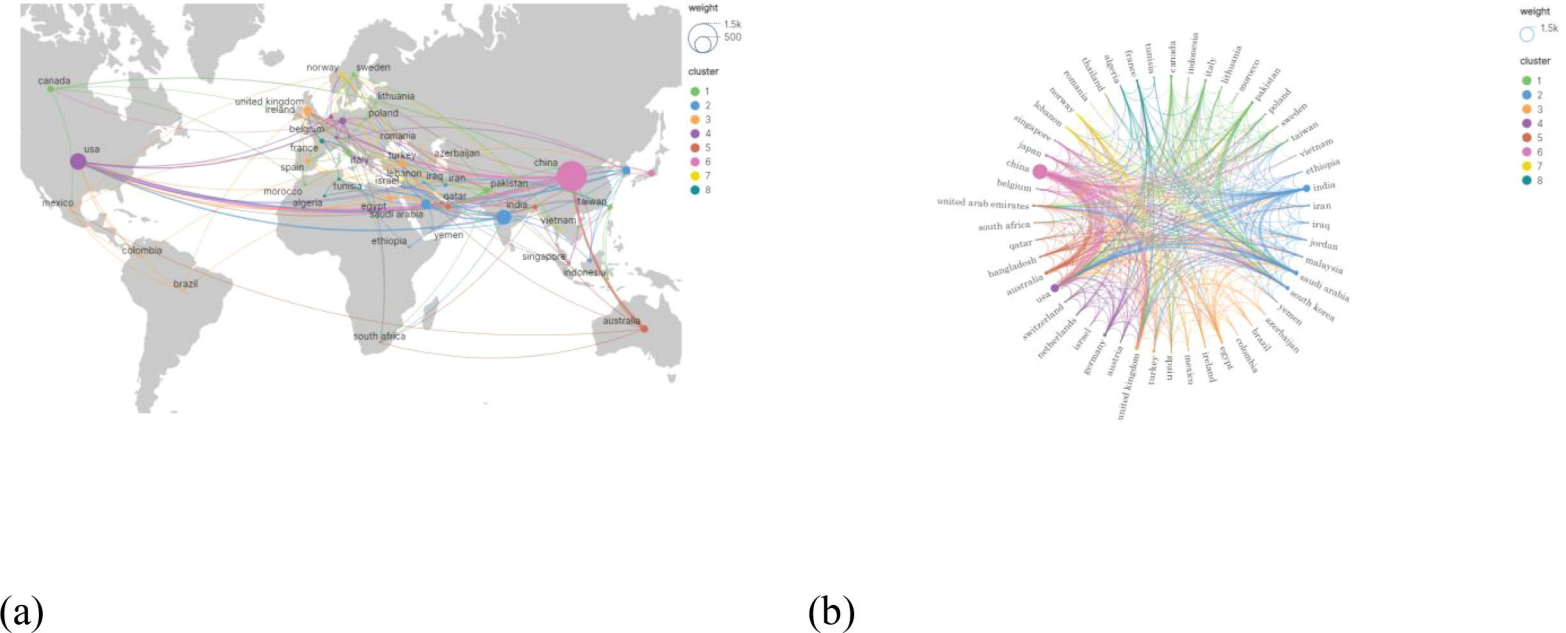
Publications and cooperation in different countries/regions of the world. **(A)** Map of the world’s countries/regions in terms of publications and collaborations in the field of TICD. (The size of the circle represents the number of articles issued. The thickness of the connecting line represents the number of collaborative communications between countries. The color of the circles represents the intensity of cooperation.). **(B)** Cooperation between countries/regions.

**Figure 4A** visually illustrates the distribution of research efforts and international collaborations in the field of TICD. The publishing countries are primarily concentrated in East Asia, and parts of the Americas, with China and the United States leading in publication volume and demonstrating frequent collaboration. **Figure 4B** further depicts international cooperation patterns, showing that China and the United States have the most extensive collaborations, engaging in research partnerships with multiple countries. The United Kingdom, Saudi Arabia, and India also exhibit significant international cooperation.

Chinese scholars have published the highest number of papers, totaling 1494; however, the average number of citations per paper is 10.67, indicating a moderate citation impact. The United States and India follow, with 444 and 352 publications, respectively. Australia has the highest average citation count per paper at 23.8, with 92 papers cited 2190 times. The United States ranks second, with 444 publications receiving 9760 citations, resulting in an average of 21.98 citations per paper. The publication volume and citation statistics for the top 10 publishing countries are presented in **Table 1**.

**Table 1:** Publications and citations of the top 10 countries.

Country	Documents	Citations	Citations/ documents
China	1494	15941	10.67
USA	444	9760	21.98
India	352	1752	4.98
Saudi Arabia	154	1327	8.62
England	120	1944	16.20
South Korea	111	821	7.40
Australia	92	2190	23.80
Germany	88	1721	19.56
Turkey	76	902	11.87
Japan	72	425	5.90

### 3.4 Analysis of research institutions

**Figure 5:**
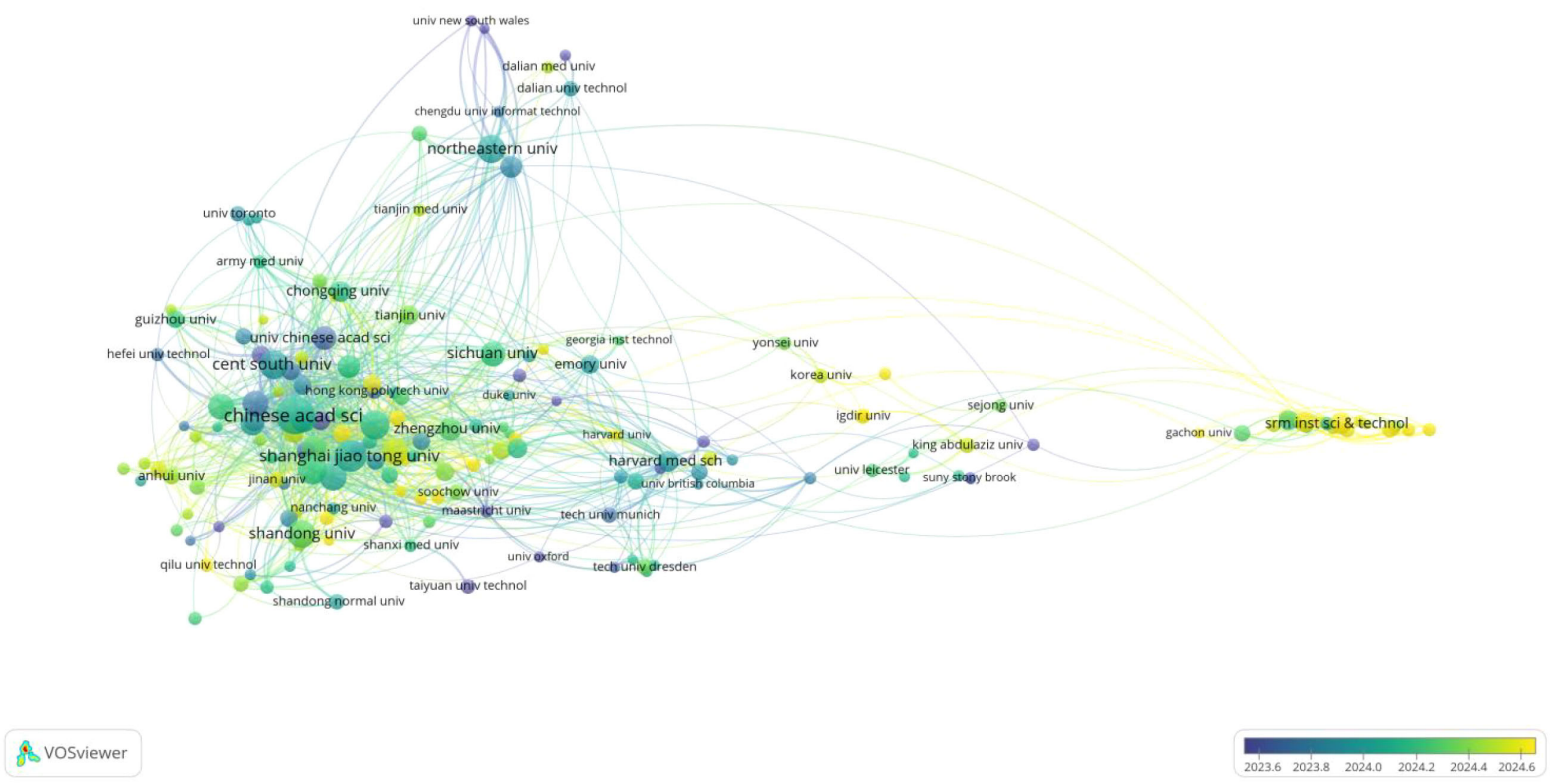
Inter-institutional collaboration status. Each node represents an academic institution, with node size indicating the number of publications. Node and link colors are mapped to the time period of publication (blue: 2009–2014, green: 2014–2019, yellow: 2019–2024). Line thickness reflects the strength of collaborative relationships between institutions.

**Figure 5** illustrates the co-authorship network among institutions that have published more than eight papers, with a total of 175 institutions meeting this threshold. The top five institutions in terms of publication volume are the Chinese Academy of Sciences (92), Shanghai Jiao Tong University (64), Central South University (55), Sun Yat-sen University (55), and Fudan University (54), as showned in **Table 2**.

**Table 2:** Publications and citations of the top 10 institutions.

Institution	Documents	Citations	Citations/ documents
Chinese academy of Science	92	1032	11.22
Shanghai Jiao Tong University	64	587	9.17
Central South University	55	833	15.15
Sun Yat-sen University	55	626	11.38
Fudan University	54	355	6.57
Shandong University	50	306	6.12
Northeastern University	49	953	19.45
Zhejiang University	47	430	9.15
Beihang University	44	504	11.45
Southern Medical University	44	369	8.39

From the perspective of publication quality (with a minimum threshold of five publications), the Vanderbilt University (USA) has the highest average citations per paper (176.85), despite having published only 13 papers with 2299 citations. It is followed by ShanghaiTech University (China) with an average of 97.13 citations per paper, East China Normal University (China) (59.38), University of Lübeck (Germany) (52.63), and The Hong Kong University of Science and Technology (Hongkong, China) (50.73).

The institutional collaboration network forms 11 clusters, reflecting the strength of collaborative relationships. Domestic collaborations are frequent, though international partnerships are also significant. The vast majority of clusters include Chinese institutions, which are predominantly universities. Collaborations among Chinese institutions are fairly frequent, and the scope of domestic cooperation is not limited by geographical location, with extensive partnerships established. Some institutions have collaborated with research-powerful organizations in other countries, such as harvard university(USA), University of New South Wales(Australia), University of Lübeck(Germany) and University New South Wales (Australia). European countries engage in more extensive international collaborations. For instance, Harvard Medical School has established extensive partnerships with a large number of institutions from various other countries, such as Memorial Sloan Kettering Cancer Center(USA), University of Leeds(The United Kingdom), German Cancer Research Center and RWTH Aachen University (Germany).

Different colors in **Figure 5** represent the temporal evolution of institutional research on this topic. The Chinese Academy of Sciences and Northeastern University (USA) were among the earliest institutions to conduct research in this area (June 2023). During the period from April to June 2024, institutions with significant publication output included Princess Nourah Bint Abdulrahman University(Saudi Arabia), SRM Institute of Science & Technology(India), Chitkara University(India), Igdir University(Turkey) etc. Research on this topic by these institutions started relatively late.

### 3.5 Bibliometric analysis of authors

**Figure 6:**
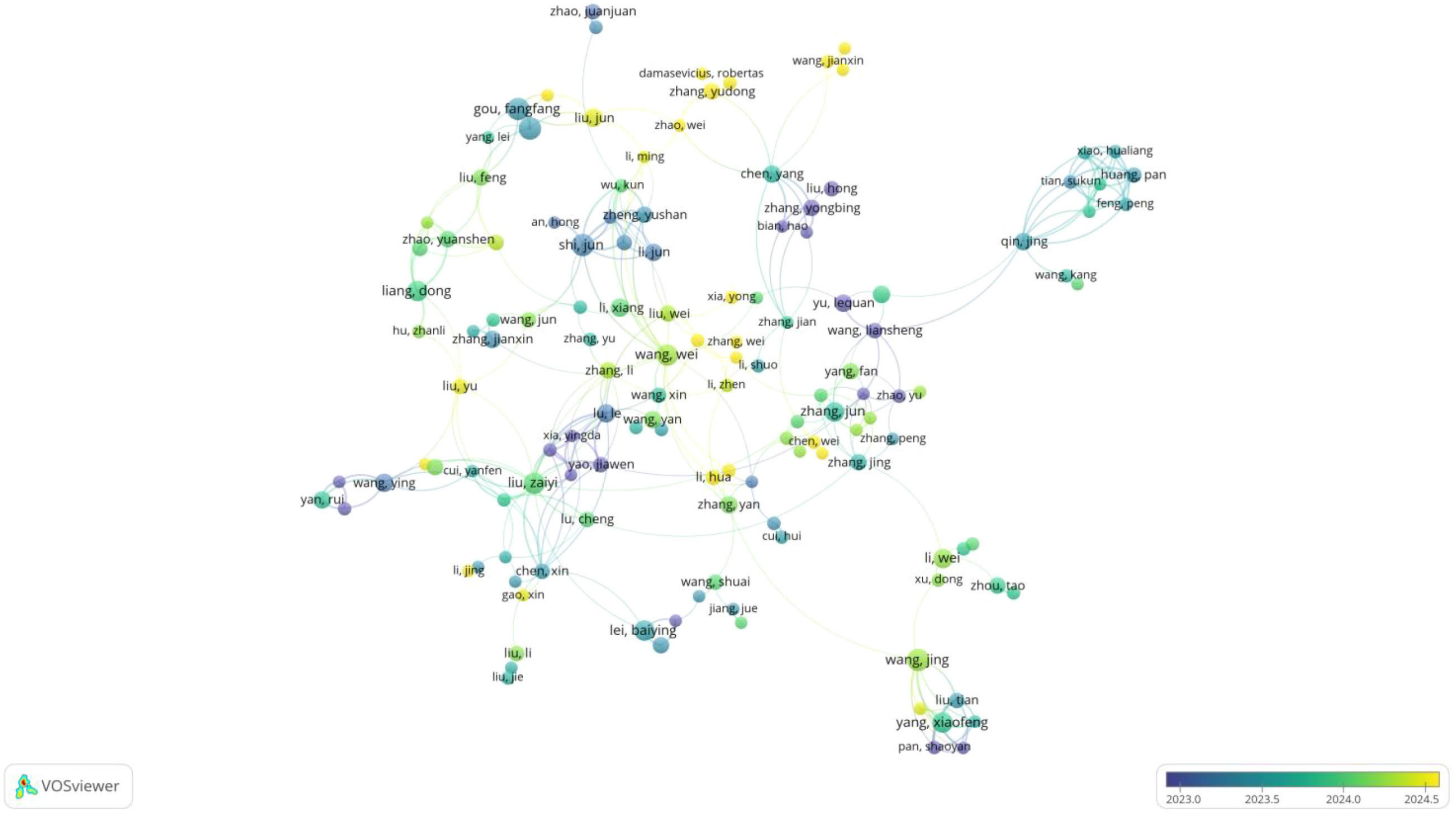
Visualization of co-authorship.

We constructed an author co-authorship network based on authors with more than five publications(**Figure 6**), identifying 194 authors within this threshold. The largest connected network comprises 135 authors, accounting for 69.59% of the total, indicating that more than half of the researchers collaborate with others. The collaboration network consists of 16 clusters, with the largest cluster containing 18 authors and the smallest containing 2 authors.

Among the most collaborative researchers, Li, Chen, Grzegorzek, Marcin, and Sun, Hongzan exhibit the highest total link strengths, with 50, 49, and 46 links, respectively. From a temporal perspective, early contributors to this field include Zhang yongbin, Yu lequn, and Yao jiawen, who were active as early as 2023. Between 2024 and 2025, notable researchers included Kuang hulin, Zhang yudong, and Liu jun have more recently focused on this field.

The top 10 most prolific scholars in this area, the majority of whom are from China. Pacal, ishak lead in publication volume, with 16 papers. They are followed by Shi, jun (15 papers), Gou, fangfang (15 papers), and Wu, jia (15 papers) and Wang, jing(15 papers). In terms of research impact, Tang, yucheng, Bian, hao, and Wang, yifeng have the highest average citation counts per paper, with 379, 140, and 137.4 citations, respectively.

### 3.6 Analysis of journals and cited journals

Among the 818 sources, 104 have published more than five papers on the application of TICD. **Table 3** lists the top 11 sources in terms of publication volume The majority of these journals belong to the JCR Q1. The top three journals in terms of publication volume are Biomedical Signal Processing and Control, IEEE Access, and Scientific Reports, which have published 181, 123, and 108 papers, accounting for 6.19%, 4.24%, and 3.69% of the total, respectively. Among the top 11 sources, Medical Image Analysis has the highest total citations (3016) and the highest average citations per paper (62.83), making it the most influential journal in this field.

**Table 3:** Publications an d citations of the top 11 sources.

Sources	IF (2024)	JCR (2024)	Documents	Citations	Citations/ documents
Biomedical Signal Processing and Control	4.9	Q2	181	1356	7.49
Ieee Access	3.6	Q2	123	681	5.54
Scientific Reports	3.9	Q1	108	661	6.12
Ieee Journal of Biomedical and Health Informatics	6.8	Q1	60	750	12.50
Medical Image Analysis	11.8	Q1	48	3016	62.83
Medical Physics	3.2	Q1	47	210	4.47
Diagnostics	3.3	Q1	45	728	16.18
Expert Systems With Applications	7.5	Q1	44	459	10.43
Frontiers in oncology	3.3	Q2	43	148	3.44
Medical Image Computing and Computer Assisted Intervention - Miccai	/	/	41	1514	36.93
International Journal of Imaging Systems and Technology	2.5	Q2	38	105	2.76

**Figure 7:**
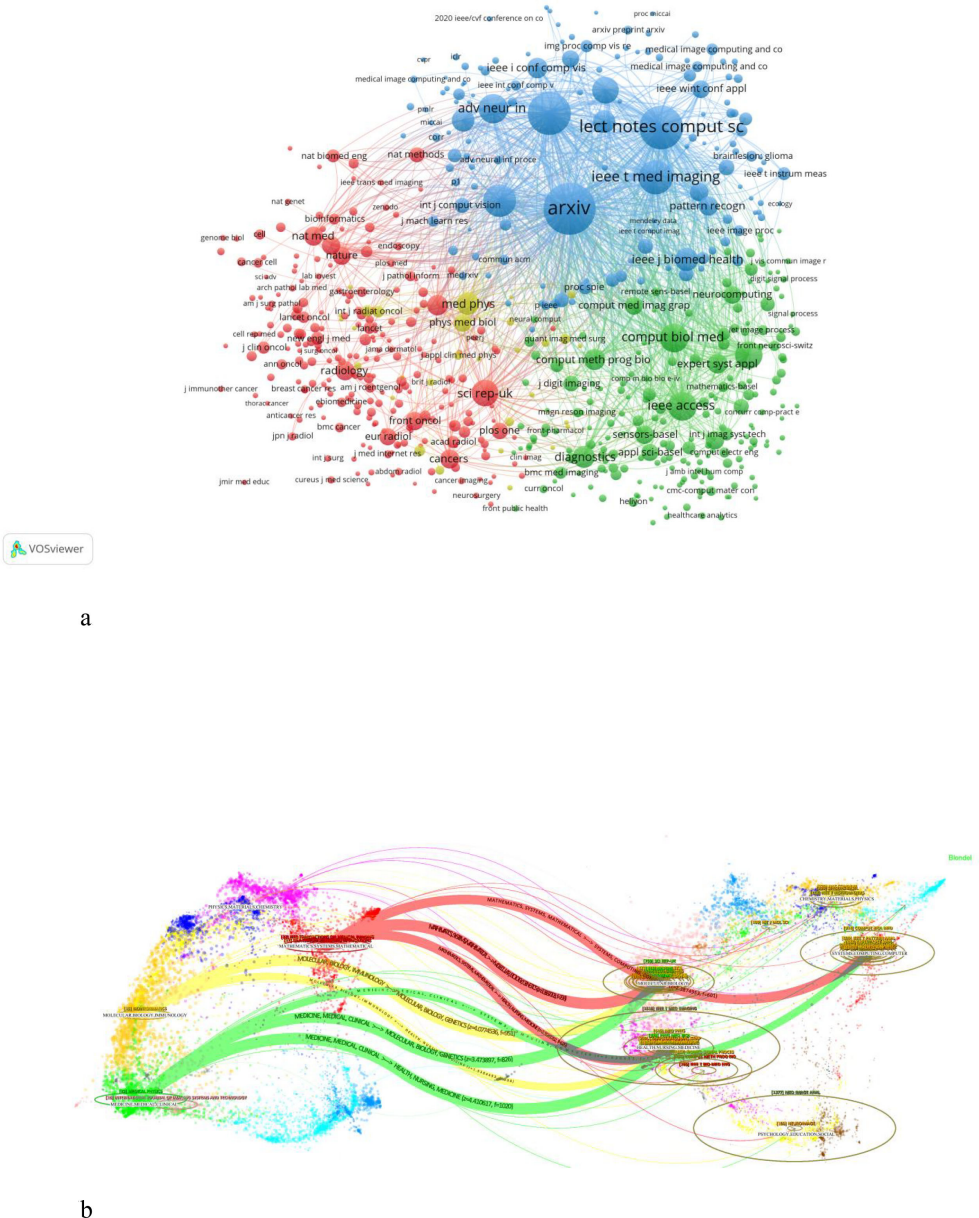
**(A)** Co-citation relationships between journals. **(B)** A dual-map overlap of journals on TICD.

In total, the collected papers cite references from 14336 different journals. Setting the threshold for cited journal occurrences at more than 20, we constructed a co-citation network of cited journals (**Figure 7A**). The top three cited sources are arXiv (9715 citations), Lecture Notes in Computational Science and Engineering (6237 citations), and Conference on Computer Vision and Pattern Recognition (CVPR) (5981 citations). The cited sources form four distinct clusters:The red cluster is primarily related to radiology research; The green cluster is mainly associated with the computer medical imaging; The blue cluster focuses on the intersection of computer science and biomedical fields; The yellow cluster is associated with medical physics.

Using the overlay maps function in CiteSpace, we visualized the citation relationships between citing and cited journals (**Figure 7B**). This analysis reveals the academic disciplines where Transformer-related oncology research is published and referenced. Citing journals mainly belong to four disciplines: 1) Medicine, medical, clinical; 2) Molecular biology, immunology; 3)Mathematics, systems, mathematical; 4) Physics, materials, chemistry. Cited journals are primarily concentrated in five disciplines: 1)Psychology, education, social sciences; 2) Health, nursing, medicine; 3)Molecular biology; 4) Systems, computing, computer science;5) Chemistry, materials, physics.

### 3.7 Co-occurrence analysis of keywords

**Figure 8:**
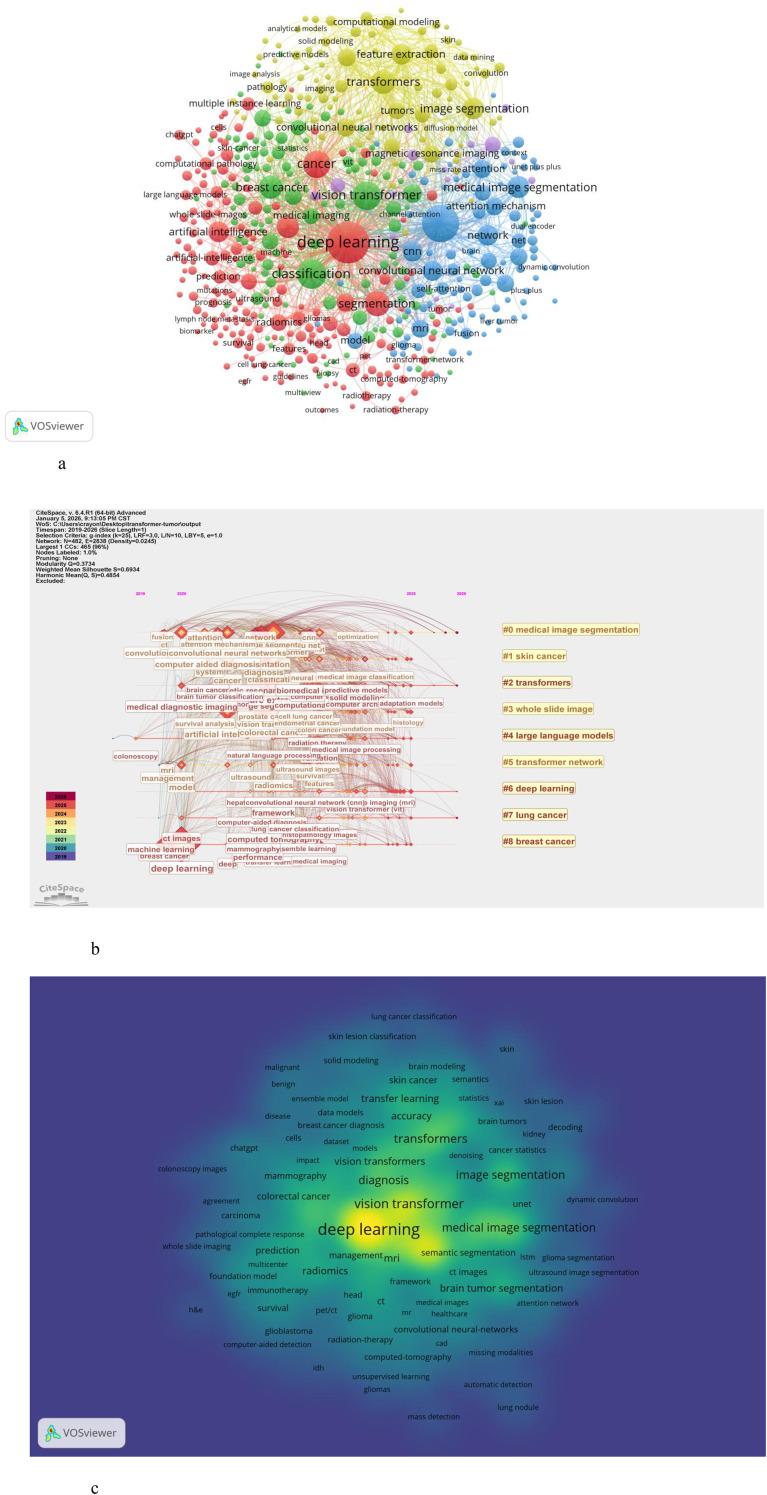
**(A)** Co-occurrence analysis of keywords. **(B)** Temporal evolution of keywords. **(C)** The density map of TICD research. The size of word, the size of round, and the opacity of yellow is positively related to the co-occurrence frequency.

We selected keywords that appeared more than five times for visualization analysis using VOSviewer (**Figures 8A,C**). Among the 6368 keywords, 508 met the selection criteria. The most frequently occurring keywords include deep learning (843), followed by transformer (633), classification (345), vision transformer (326), and cancer (289). The cancer types that have received the most attention from scholars are shown in **Table 3**, with breast cancer being the most studied (371), followed by brain cancer, skin cancer and lung cancer. See **Table 4** for details.

**Table 4:** The frequency of keywords related to different types of diseases.

Types of cancer	Frequency of keyword
breast cancer	371
brain cancer	353
skin cancer	198
lung cancer	181
colorectal cancer	143
liver cancer	69
cervical cancer	65
gastric cancer	27
carcinoma	16
thyroid cancer	15
pancreatic cancer	11
kidney cancer	11
head and neck cancer	10
oral cancer	10
osteosarcoma	10
laryngeal cancer	7

Using CiteSpace, we generated a keyword evolution map, which retains eight clusters(**Figure 8B**). The eight clusters collectively focus on deep learning (DL) applications, especially vision transformers (ViT) and convolutional neural networks (CNNs), in medical oncology, centering on cancer diagnosis, segmentation, and prognosis via medical imaging and computational pathology.

Clusters 0, 2, 7, and 8 focus on DL-based medical image analysis for lung and breast cancer, emphasizing multi-scale feature fusion, boundary enhancement, and ViT-CNN integration to improve diagnostic accuracy via CT and mammographic images.Clusters 1, 5, and 6 specialize in breast cancer research, covering BI-RADS classification, neoadjuvant chemotherapy response assessment, and survival prediction, with innovations in ViT-CNN modules and attention mechanisms.Clusters 3 and 4 extend to computational pathology and cross-modal AI, integrating natural language processing (NLP) with digital pathology for thyroid cancer/lymphatic metastasis detection (Cluster 3) and applying large models (SAM, LLMs) to pulmonary nodule segmentation and colorectal neoplasm staging (Cluster 4).Overall, these clusters reflect a paradigm shift toward transformer-augmented DL in oncology, featuring multi-modal data integration and translational focus. ViT-CNN hybrid architectures and large foundation models are core enablers for advancing AI-driven precision diagnosis and prognosis across diverse cancers.

The evolution of keywords over time also reveals research trends. Terms such as transformer, medical image segmentation and multiple instance learning appeared earlier, primarily between 2023 and 2024. As research progressed, scholars expanded into various application areas, with keywords like accuracy, explainable AI, Medical Image Analysis and foundation model becoming more prevalent after 2025.

If certain keywords are concentrated within a specific period, they can be considered burst terms, reflecting different stages of development within a field. Using CiteSpace, we identified the top 11 most prominent burst keywords from studies on TICD (**Table 5**). The most intense burst term was “attention” in 2021, followed by “survival analysis” in 2019. More recently, scholars have focused on computer-aided diagnosis and radiomics, indicating emerging research directions.

**Table 5:** The top 11 burst words of research on TICD.

Keywords	Year	Strength	Begin	End
attention	2021	5.35	2021	2023
survival analysis	2021	5.02	2021	2023
computer aided diagnosis	2021	3.21	2021	2023
whole slide image	2022	3.02	2022	2023
visual transformer	2022	2.95	2022	2023
cancer	2021	2.91	2021	2022
skin lesion	2023	2.89	2023	2024
pulmonary nodules	2022	2.63	2022	2023
liver tumor segmentation	2022	2.52	2022	2023

The columns “Begin” and “End” represent the start and end years of the burst detection for each keyword, respectively. The “Strength” column indicates the intensity of the keyword burst during the corresponding time period.

### 3.8 Analysis of articles and references

Among the 2923 papers we screened, 54 have been cited more than 100 times. Among them, the highly cited literatures are from *Medical Image Analysis:* Chen, XX et al. (30) reviewed the latest advances and contributions of unsupervised and semi-supervised deep learning in medical image analysis, discussing the main technical challenges and potential solutions is the most cited, with 310 citations since its publication in 2022. Shamshad, F et al. (31) explored the application of transformers in medical imaging, cited 206 times.Wang, XY et al. (32) trained an unsupervised transformer-based model for tissue pathology image classification and performed performance comparisons.Most of the top 10 cited papers are related to medical imaging, such as medical image segmentation (33), medical image classification (34, 35), and pathology image detection (36). The significant potential of transformers for single-cell annotation has also been recognized (37), as well as the specific applications to certain clinical disciplines, such as gynecology (38, 39).

**Figure 9:**
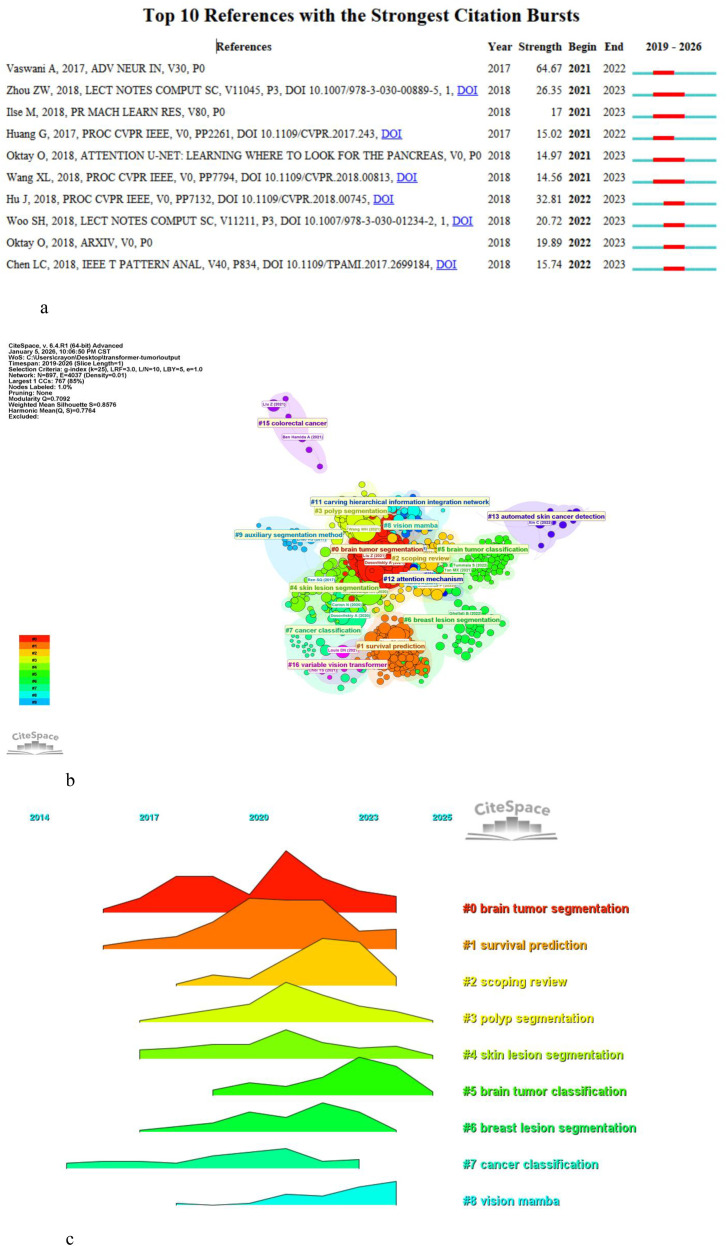
**(A)** Top 10 references cited in burst. **(B)** Co-citation analysis of references. **(C)** Timeline diagram of references.

**Figure 9A** shows the burst citation papers. After 2018, there was a surge in burst citations, indicating that the application of TICD began to rapidly develop after 2018. The paper with the highest burst intensity is Vaswani, A et al. (4), who proposed the transformer architecture in 2017.

In total, all the papers cited 65496 references, with 20 papers having citation counts of 20 or more. Co-citation analysis was conducted using CiteSpace (**Figure 9B**). The cited papers form eight clusters. The top-ranked paper by citation count is “An Image is Worth 16x16 Words: Transformers for Image Recognition at Scale” by Dosovitskiy A et al. (40), with 877 citations.The paper pioneered the application of Transformers in image recognition by tokenizing images into 16×16 patches as input sequences, breaking the inherent locality limitation of convolutional neural networks (CNNs) and enabling effective global feature modeling at scale. The second from Liu, Z (41), with 775 citations, which proposed the Swin Transformer, introducing hierarchical architecture and shifted windows to address the computational inefficiency of vanilla Transformers in visual tasks, facilitating efficient and fine-grained feature learning for computer vision.The third is Chen, Jieneng (2021, arXiv, V0, P0) (42), with 501 citations.He presented TransUNet, integrating Transformers as strong encoders for global context extraction with U-Net’s decoder for high-resolution local detail recovery, effectively overcoming the trade-off between global dependency modeling and precise localization in medical image segmentation.

We used the timeline view to visually display the categorization and publication years of the cited references (**Figure 9C**). Most of the cited papers were published between 2017 and 2024. The topic brain tumor segmentation has been continuously cited for the longest period, while vision mamba had a shorter citation duration.

## 4 Discussion

Since the transformer architecture was introduced in 2017, scholars have continuously explored its potential in various fields (43–46), leading to significant advances in oncology, particularly in diagnosis (47, 48). Bibliometric analysis can assess authors, institutions, countries, and references from the SCIE database, and through tools like CiteSpace and VOSviewer, the findings can be visualized. This approach provides readers with a global perspective on the evolving research dynamics of transformer applications in oncology and offers insights into future trends. This study uses bibliometric analysis and visualization to explore the applications and developments in the field from 2017 to the present and to forecast future research directions.

Before 2022, the development of TICD was slow, with fewer than 50 papers published annually. However, starting in 2022, the number of publications surged, continuing to show strong growth. It is expected that 85 early access papers will be published by 2026, indicating that the field is flourishing and attracting widespread attention from researchers (49, 50). The U.S. and China dominate the number of published papers, which also reflects an uneven distribution of global research efforts. However, Australia and Germany have the highest average citation rates per paper, suggesting a discrepancy between the number of papers published and their impact. International collaboration is prominent, with China and the U.S. collaborating extensively with other countries, highlighting that cross-border exchange is beneficial for scientific progress.

The institutions that publish the most papers in this field are mostly based in China, such as Chinese Academic Science, Shanghai Jiao Tong University, and Central South University. Influential institutions include Northeastern University, Central South University and Beihang University, with research foci in interdisciplinary computer science applications (51–53), radiology, nuclear medicine, and medical imaging (54, 55), and medical informatics (56). While Chinese institutions produce a high volume of publications, they tend to have lower average citation rates, which could be due to certain Chinese research policies requiring rapid completion of research goals. As a result, Chinese researchers may publish more papers, but not have sufficient time to produce high-quality work. It may also be because such studies often focus on the peripheral extensions of mature fields, rather than addressing the core scientific issues or technical bottlenecks within the field. Peripheral studies, failing to provide key support for the development of the field, may only be mentioned by a small number of researchers in the same direction, resulting in a low average number of citations per paper. Additionally, Zipf’s law might be at play, where 20% of authors produce 80% of the high-quality papers, and due to a larger base of researchers in China, the average citation rate is lower.

Researchers in this field engage in broad collaboration, with more than half of the authors collaborating with others. The three authors with the most collaborations have an average of 48 co-authored papers. This indicates a high level of collaboration, likely because this field lies at the intersection of medicine and computer science, attracting a diverse range of researchers. Such collaboration helps uncover novel research directions and broaden research perspectives.

The journals in which the most influential research in this field is published are mostly in the JCR Q1 category, indicating that the field’s research outputs are generally found in high-impact journals. The most frequently cited journals are preprints and conference papers, which highlights that cutting-edge research is often first presented in these formats. This suggests that academic conference participation plays a crucial role in staying up-to-date with the latest trends and generating innovative research ideas, thereby fostering scientific advancement.

The main research directions in this field focus on the application of transformer-based algorithms in tumor-assisted diagnosis, growth prediction. Key methods include image segmentation and classification, association prediction, and motion simulation (57–60).Around 2020, from the perspective of task implementation, Transformers were mainly used for feature fusion (61). Since 2021, research related to medical images segmentation has begun to surge (62–64). Subsequently, the research has expanded to cover multiple aspects centered on Vision Transformers (ViTs), including object detection, medical image classification, medical image enhancement (65). Additionally, it also includes research directions such as clinical report generation, and clinical fact retrieval (66–68). From the perspective of performance comparison, early studies mostly compared Transformers with simple deep learning network architectures such as convolutional neural networks (CNNs) (69). Since 2022, different studies have focused on the performance improvement of Transformer-based models in single tasks (e.g., image segmentation) and multiple complex tasks (e.g., feature extraction and prediction) (68, 70, 71). In contrast, the research trend since 2023 has tended to use multimodal Transformers to accomplish various tasks (72–74).

Among all the 2923 publications, only a small number of studies have explored TICD from the perspective of clinical trial applications. Specifically, there are 3 randomized controlled trials (RCT) focusing on TICD that were published in 2023 (75–77). One research focused on combing transformer with U-net for predicting measurement-guided volumetric dose (MDose) of performing pre-treatment patient-specific quality assurance for volumetric modulated arc radiotherapy (VMAT) (78). The other two studies employed transformer and other deep learning model to predict lymph node (LN) metastasis in lung and gastric cancer (75, 77). Another article published in 2024 develops and validates the Transformer-based T-BEHRT model (with doubly robust estimation) to estimate antihypertensives’ effect on incident cancer risk via EHRs, showing it outperforms benchmarks and aligns with RCT results (78).

The studies all confirmed the superior performance of applying transformer-based deep learning model to capture micro- and micro-structures in cancer research, especially the revolutionized introduction of self-attention mechanism. Transformer was first introduced to handle sequential inference tasks in natural language processing (NLP), which showed remarkable capabilities in capturing long-term dependencies of sequential data with stacked self-attention layers (79). The attention mechanism allows to (soft-)search for the most relevant information and encourage the model to predict specific features based on the context vectors associated with all the previous generated information (80). In this case, the model can process information with “memory” and select the most relevant feature in multi-source heterogeneous data.

While there have been notable research achievements in the application of TICD, most studies use specific datasets or focus on particular situations. Based on the aforementioned analysis, we propose targeted and actionable future research directions. From a technical perspective, it is essential to develop lightweight Transformer models tailored for small-sample cancer data and optimize the attention mechanism to enhance the capability of capturing subtle lesion features. Regarding clinical translation, establishing a standardized workflow encompassing “algorithm development - clinical pilot - multi-center validation” is a priority, along with promoting the interface adaptation between Transformer models and clinical diagnosis and treatment systems. From a methodological standpoint, reproducibility reporting standards for medical AI research should be formulated, mandating the disclosure of core codes, data descriptions, and training parameters. In terms of application expansion, efforts should be directed toward exploring the application of Transformers in the diagnosis and treatment of rare cancers, as well as conducting multi-dimensional analyses of cross-modal data fusion, including imaging, pathology, and genetics.

## 5 Limitations

The manuscript does have some limitations. Firstly, while bibliometric methods provide a global perspective on the development, hotspots, and gaps in a research field, they overlook the finer details that can be captured from a more micro-level perspective. Secondly, bibliometric research may have a delay in reflecting the most cutting-edge studies, which could lead to biases when predicting future trends. Thirdly, this study only included English-language literature. Although a large proportion of internationally published research is presented in English, there remains a possibility that we may have overlooked some important studies published in other languages. Furthermore, given that the research objective is to provide a descriptive overview of the topic at a macroscopic level, no strict indicator-based screening was applied during literature inclusion. Accordingly, statistical tests and quantitative network metrics were not performed, as these are not standard requirements for descriptive bibliometric research. Future studies may adopt a combination of quantitative and qualitative approaches to further refine the research.

## 6 Conclusions

The application of TICD holds immense potential for tumor diagnosis and has garnered significant attention from researchers. This field is in a stage of rapid development, with China and the United States being the leading nations in research efforts. There is substantial international collaboration, but there is a need to focus on the generalizability and applicability of research findings to ensure successful translation into clinical practice.

## Data Availability

The original contributions presented in the study are included in the article/supplementary material. Further inquiries can be directed to the corresponding authors.
